# Long-term specific IgG response to SARS-CoV-2 nucleocapsid protein in recovered COVID-19 patients

**DOI:** 10.1038/s41598-021-02659-4

**Published:** 2021-12-01

**Authors:** Jira Chansaenroj, Ritthideach Yorsaeng, Nawarat Posuwan, Jiratchaya Puenpa, Nasamon Wanlapakorn, Natthinee Sudhinaraset, Manit Sripramote, Piti Chalongviriyalert, Supunee Jirajariyavej, Phatharaporn Kiatpanabhikul, Jatuporn Saiyarin, Chulikorn Soudon, Orawan Thienfaidee, Thitisan Palakawong Na Ayuthaya, Chantapat Brukesawan, Chintana Chirathaworn, Duangnapa Intharasongkroh, Dootchai Chaiwanichsiri, Mila Issarasongkhram, Rungrueng Kitphati, Anek Mungaomklang, Pijaya Nagavajara, Yong Poovorawan

**Affiliations:** 1 Center of Excellence in Clinical Virology, Faculty of Medicine, Chulalongkorn University, Bangkok, Thailand; 2 Division of Academic Affairs, Faculty of Medicine, Chulalongkorn University, Bangkok, Thailand; 3 Medical Service Department, Bangkok Metropolitan Administration, Bangkok, Thailand; 4 Taksin Hospital, Medical Service Department, Bangkok Metropolitan Administration, Bangkok, Thailand; 5 Charoenkrung Pracharak Hospital, Medical Service Department, Bangkok Metropolitan Administration, Bangkok, Thailand; 6 Klang General Hospital, Medical Service Department, Bangkok Metropolitan Administration, Bangkok, Thailand; 7 Sirindhorn Hospital, Medical Service Department, Bangkok Metropolitan Administration, Bangkok, Thailand; 8 Ratchaphiphat Hospital, Medical Service Department, Bangkok Metropolitan Administration, Bangkok, Thailand; 9 Public Health Center 28, Health Department, Bangkok Metropolitan Administration, Bangkok, Thailand; 10 Public Health Center 26, Health Department, Bangkok Metropolitan Administration, Bangkok, Thailand; 11 Tropical Medicine Cluster, Faculty of Medicine, Chulalongkorn University, Bangkok, Thailand; 12 National Blood Center, Thai Red Cross Society, Bangkok, Thailand; 13 Institute for Urban Disease Control and Prevention, Department of Disease Control, Ministry of Public Health, Bangkok, Thailand; 14 Office of the Permanent Secretary for the Bangkok Metropolitan Administration, Bangkok, Thailand

**Keywords:** Infectious diseases, Virology

## Abstract

This study monitored the long-term immune response to severe acute respiratory syndrome coronavirus (SARS-CoV)-2 infection in patients who had recovered from coronavirus disease (COVID)-19. Anti-nucleocapsid immunoglobulin G (anti-N IgG) titer in serum samples collected at a single (N = 302) or multiple time points (N = 229) 3–12 months after COVID-19 symptom onset or SARS-CoV-2 detection in respiratory specimens was measured by semiquantitative chemiluminescent microparticle immunoassay. The 531 patients (966 specimens) were classified according to the presence or absence of pneumonia symptoms. Anti N IgG was detected in 87.5% of patients (328/375) at 3 months, 38.6% (93/241) at 6 months, 23.7% (49/207) at 9 months, and 26.6% (38/143) at 12 months. The anti-N IgG seropositivity rate was significantly lower at 6, 9, and 12 months than at 3 months (*P* < 0.01) and was higher in the pneumonia group than in the non-pneumonia/asymptomatic group at 6 months (*P* < 0.01), 9 months (*P* = 0.04), and 12 months (*P* = 0.04). The rate started to decline 6–12 months after symptom onset. Anti-N IgG sample/cutoff index was positively correlated with age (r = 0.192, *P* < 0.01) but negatively correlated with interval between symptom onset and blood sampling (r =  − 0.567, *P* < 0.01). These findings can guide vaccine strategies in recovered COVID-19 patients.

## Introduction

Coronavirus disease 2019 (COVID-19) has a broad spectrum clinical manifestations including asymptomatic, mild, severe, critical, and even fatal^1,2^. A large proportion of infected individuals are asymptomatic or have mild symptoms but are still contagious^1^. The elderly and individuals with chronic medical conditions are more likely to become severely ill from COVID-19. The severe cases eventually develop acute respiratory distress syndrome, multiple organ failure, pneumonia, and cytokine storm^3^. Diagnostic testing for early identification and isolation of infected persons and timely treatment is the main strategy used to control the spread of COVID-19.

A commonly used diagnostic test for COVID-19 is a reverse-transcription (RT)-PCR assay that detects one or more viral RNA genes reflecting current or recent infection. However, RT-PCR has some limitations: it requires sample processing in a laboratory with specialized reagents, instruments, and well-trained laboratory staff; and the poor quality of nasal or nasopharyngeal swab specimens and RNA instability can lead to false-negative results. Antibody or serologic testing to detect previous or recent infection with severe acute respiratory syndrome coronavirus (SARS-CoV)-2 can aid diagnosis. The interval between symptom onset and blood sampling affects the sensitivity and specificity of serologic tests^4^. The presence of antibodies was shown to be correlated with seroprotection in small animal models^5^ and human cell lines, supporting SARS-CoV-2 antibody-based vaccine initiatives. It is currently unknown whether a positive antibody test indicates protective immunity against SARS-CoV-2 or how long humoral responses persist after natural infection.

The SARS-CoV-2 genome encodes four structural proteins including the spike glycoprotein and nucleocapsid, envelope, and membrane proteins. The nucleocapsid protein, one of the most abundant viral proteins, has a highly conserved amino acid sequence and high immunogenicity^6^. Tests that detect antibodies to SARS-CoV-2 nucleocapsid proteins are generally more sensitive than those targeting antibodies to spike glycoprotein^7,8^. Nucleocapsid protein plays a critical role in viral pathogenesis, including aggravation of lung injury by mannan-binding lectin-associated serine protease (MASP)-2–mediated complement overactivation^9^. The protein has several epitopes that stimulate B and T cell responses and are suitable for vaccine formulations^10^. Moreover, as most SARS-CoV-2 vaccines used in humans are based on the spike antigen, the detection of antibody responses against nucleocapsid protein may be useful for distinguishing between serologic responses to infection and vaccination^11,12^.

The half-life of the SARS-CoV-2 antibody response in a cohort varies according to individuals’ age, ethnicity, and symptom severity^4,13^. Immune responses against SARS-CoV-2 spike and nucleocapsid proteins are generally detectable within 1–3 weeks after infection^14,15^. SARS-CoV-2 immunoglobulin (Ig)M antibodies can be detected as early as 3–6 days after symptom onset while IgG antibodies can be detected after 8 days^16^. A study on the kinetics of the antibody response and disease severity in patients with SARS-CoV-2 infection found that more severe cases had higher levels of IgG^17^. Monitoring the persistence of anti-N IgG following natural infection is essential for estimating population immunity and guiding vaccination strategies.

This study evaluated the SARS-CoV-2 anti-N IgG seropositivity rate in a longitudinal cohort of recovered COVID-19 patients 3–12 months after symptom onset. The early antibody response at 0–3 months after infection was previously reported^18^. We also examined factors associated with the persistence of anti-N IgG responses such as age, interval between symptom onset and blood sampling, and disease severity.

## Results

### General characteristics of the study population

Demographic data of 531 individual participants (single time point donors, n = 302; multiple time point donors, n = 229) are shown in Table 1. The mean (± standard deviation) age of the participants was 37.1 ± 12.3 years (range 2–82 years) and the median age was 36 years. The male-to-female ratio was 1.03:1 (269:262). Participants were divided into two groups according to disease severity: those without pneumonia symptoms (n = 420) and those with pneumonia symptoms (n = 111). Individuals in the pneumonia group were older than those in the non-pneumonia/asymptomatic group (*P* = 0.02). The seropositivity rate did not differ significantly between groups at 3 months after symptom onset or first SARS-CoV-2 detection (Table S1). However, at 6, 9, and 12 months the rate was significantly higher in the pneumonia group (*P* < 0.05).

**Table 1:** Demographic data of participants and specimen collection in this study.

Participants	Characteristic	Symptoms		Chi-squared (*P* value)
		Without pneumonia, N = 420	With pneumonia, N = 111	
Age, years	Median age	35	39	
	Mean age (SD)	36.8 (11.9)	40.9 (13.1)	
Age, years	< 20 (%)	11/420 (2.6)	1/111 (1.0)	9.6 (0.02)
	20–39 (%)	253/420 (60.2)	56/111 (50.5)	
	40–59 (%)	132/420 (31.4)	42/111 (37.8)	
	> 59 (%)	17/420 (4.0)	12/111 (10.8)	
	Unknown (%)	7/420 (1.7)	0/111 (0.0)	
Collection	Single collection (%)	245/420 (58.3)	56/111 (50.5)	
	Multiple collections (%)	175/420 (41.7)	55/111 (49.5)	
Sex	Male (%)	209/420 (49.8)	60/111 (54.1)	1.1 (0.3)
	Female (%)	211/420 (50.2)	51/111 (45.9)	

*SD* standard deviation.

### Long-term anti-N IgG seropositivity rates

**Figure 1:**
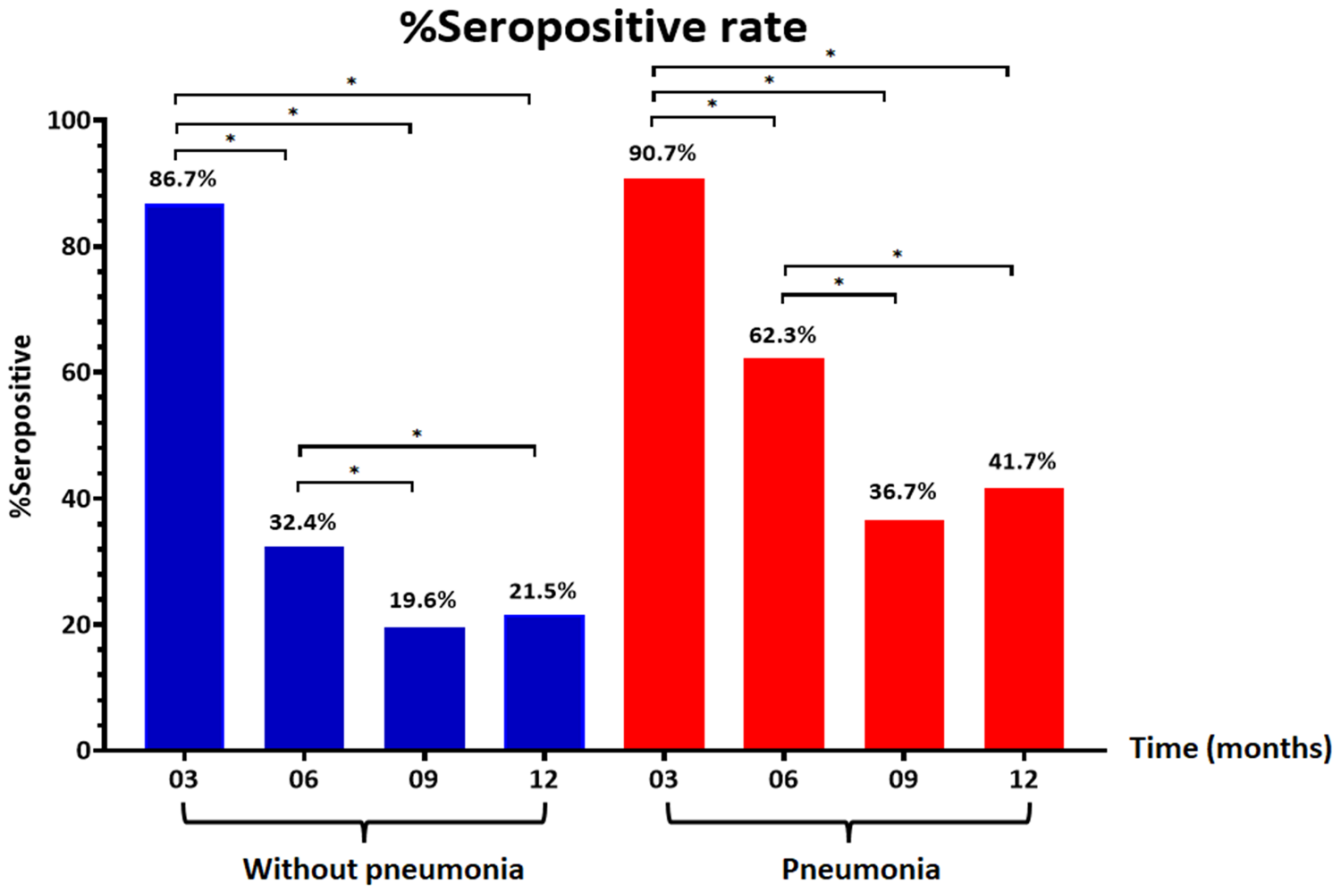
Seropositive rate of anti-nucleocapsid IgG among specimens at indicated time points after post symptom onset or first SARS-CoV-2 detection, stratified by disease severity. The cutoff was 1.4 S/C; S/C ≥ 1.4 was defined as positive and S/C < 1.4 as negative.

The seropositivity rate of anti-N IgG stratified by pneumonia/non-pneumonia group was shown in Fig. 1. In participants without pneumonia, anti-N IgG was detected in 86.7% at 3 months (260/300), in 32.4% (61/188) at 6 months, in 19.6% (31/158) at 9 months, and in 21.5% (23/107) at 12 months. In participants with pneumonia, anti-N IgG was detected in 90.7% at 3 months (68/75), in 62.3% (33/53) at 6 months, in 36.7% (18/49) at 9 months, and in 41.7% (15/36) at 12 months. The anti-N IgG seropositivity rate was significantly lower at 6, 9, and 12 months than at 3 months (*P* < 0.01) and lower at 9 and 12 months than at 6 months (*P* < 0.01) in both pneumonia and non-pneumonia group.

**Figure 2:**
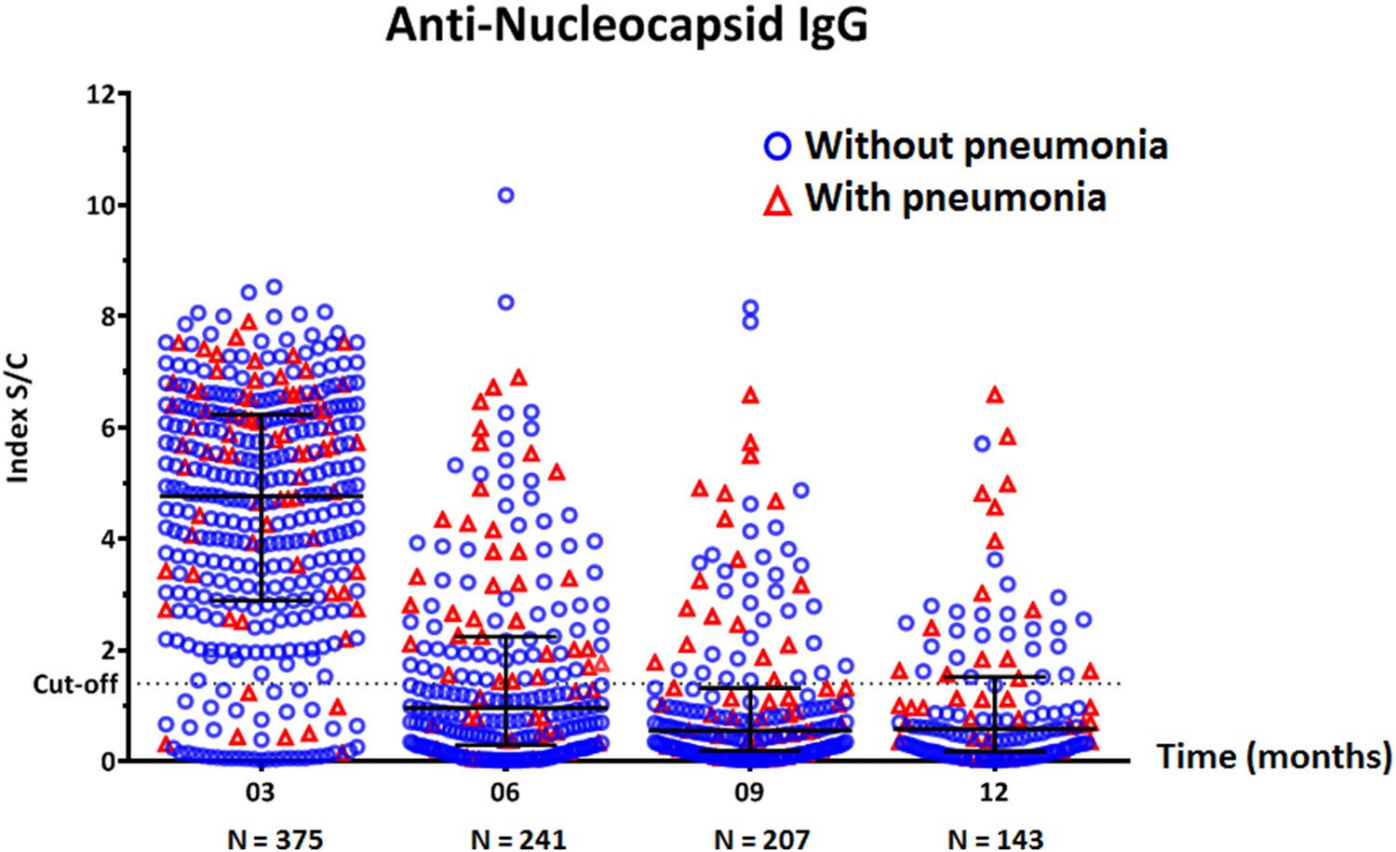
Scatter dot-plot with median and interquartile range (IQR) showing the distribution of SARS-CoV-2-anti-nucleocapsid IgG S/C index in serum samples of COVID-19 patients at 3, 6, 9, and 12 months after symptom onset or first SARS-CoV-2 detection by RT-PCR. Red triangles and blue circles represent patients with and without pneumonia symptoms, respectively.

We compared the anti-N IgG sample/cutoff (S/C) index at 3, 6, 9, and 12 months after symptom onset or first SARS-CoV-2 infection (Fig. 2). The median IgG S/C index at 3 months was 4.8 (interquartile range [IQR]: 2.9–6.2) and decreased to 1.0 (IQR: 0.3–2.2) at 6 months, 0.6 (IQR: 0.2–1.3) at 9 months, and 0.6 (IQR: 0.2–1.5) at 12 months.

**Figure 3:**
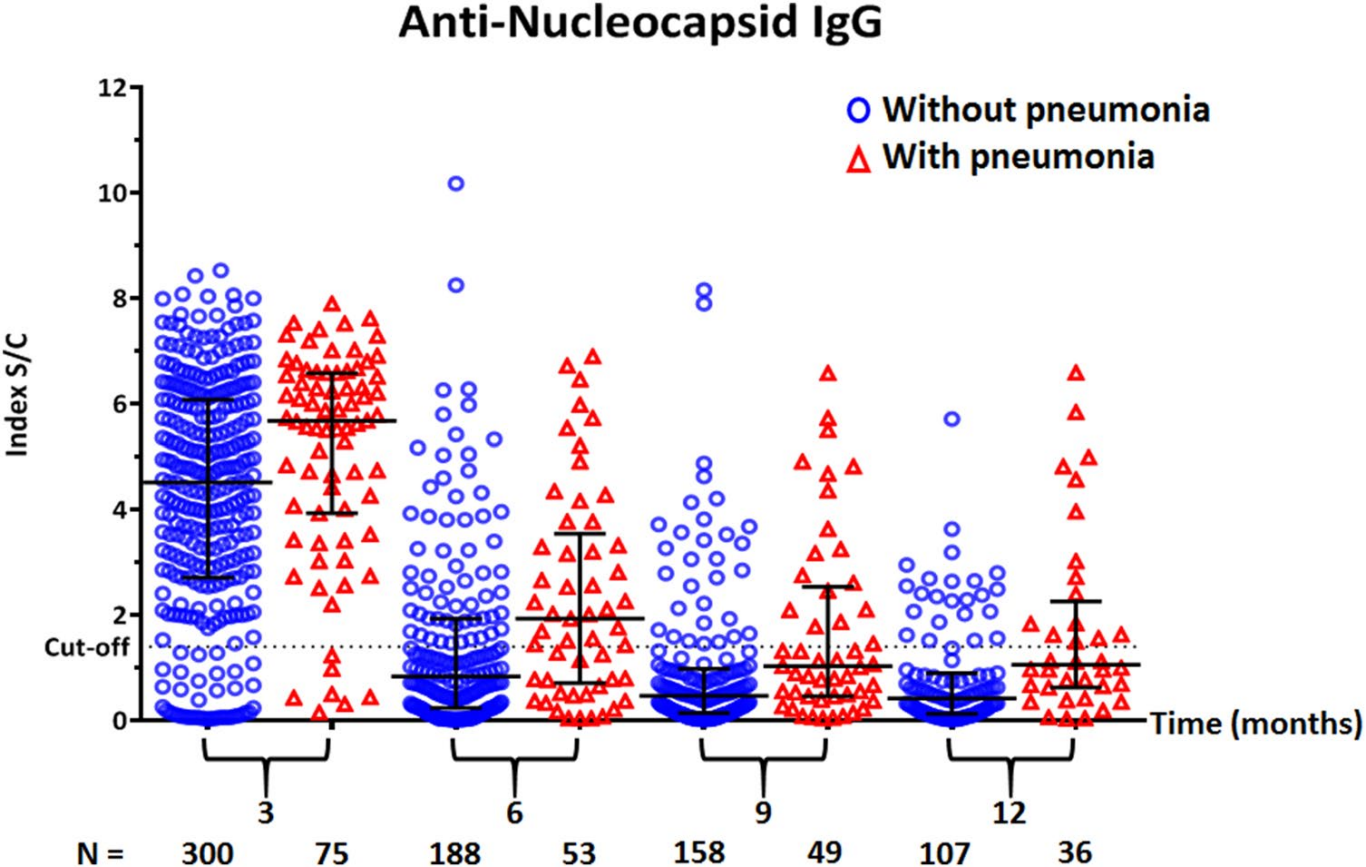
Scatter dot-plot with median and interquartile range (IQR) showing the distribution of anti-nucleocapsid IgG S/C index in serum samples of COVID-19 patients, stratified by disease severity. Red triangles and blue circles represent patients with and without pneumonia symptoms, respectively.

**Figure 4:**
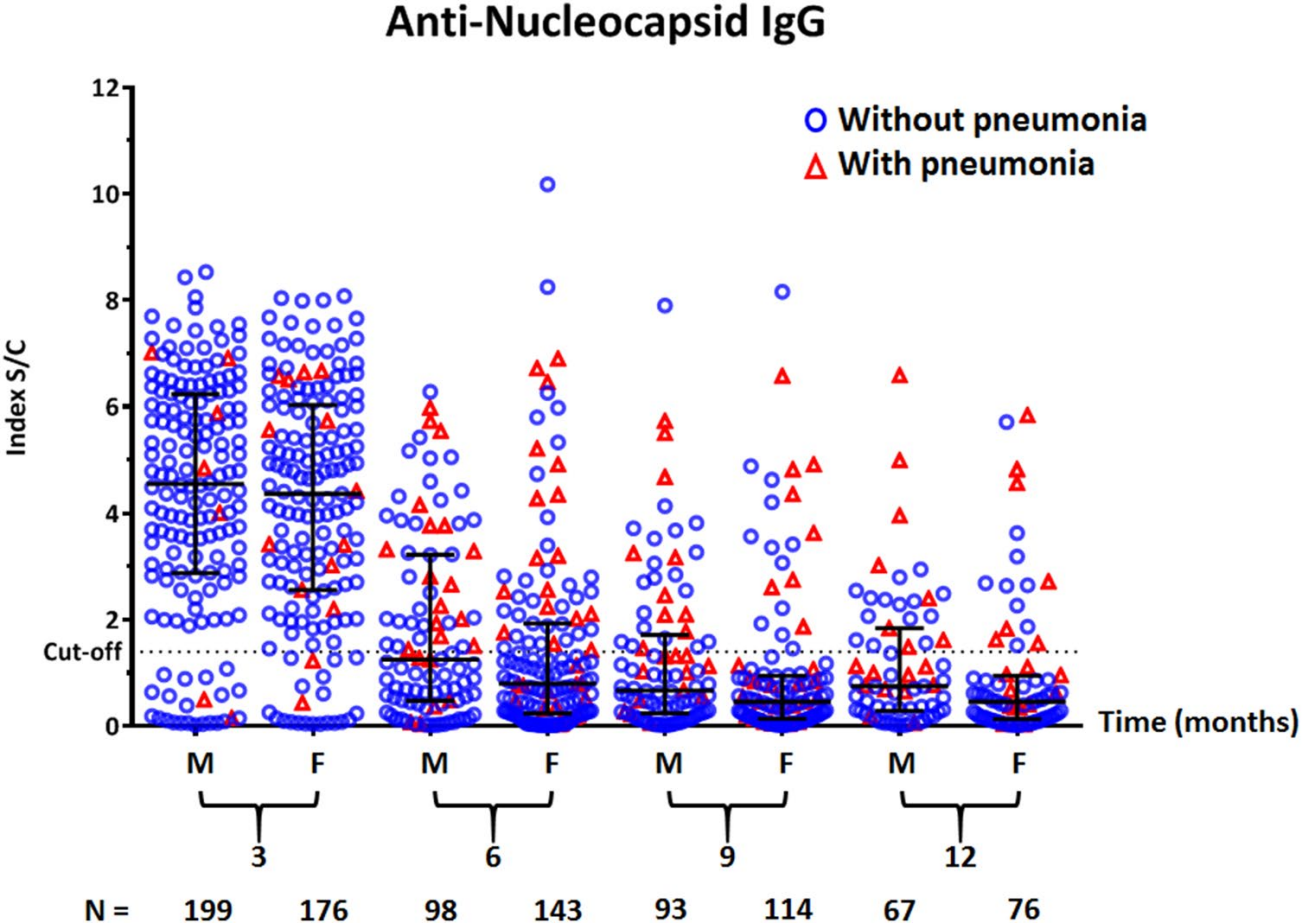
Scatter dot-plot with median and interquartile range (IQR) showing the distribution of anti-nucleocapsid IgG S/C index in serum samples of COVID-19 patients, stratified by sex (*F* female, *M* male). Red triangles and blue circles represent patients with and without pneumonia symptoms, respectively.

When classified by disease severity, the anti-N IgG S/C index decreased over time in all patients. The median IgG S/C index at 3 months tended to be higher in the pneumonia group than in the non-pneumonia/asymptomatic group (5.7 [IQR: 3.9–6.6] vs 4.5 [IQR: 2.7–6.1]), although the difference did not reach statistical significance (Fig. 3). When classified by sex, there was no difference in anti-N IgG S/C index between males and females at all time points tested (3 months, *P* = 0.45; 6 months, *P* = 0.83; 9 months, *P* = 0.90; 12 months, *P* = 0.91) (Fig. 4).

### Correlation analysis for anti-N IgG seropositivity

Pearson’s correlation coefficient was computed to assess the relationship between the anti-N IgG S/C index and interval between symptom onset and blood sampling. The IgG S/C index was negatively correlated with interval between symptom onset and blood sampling (r =  − 0.567, *P* < 0.01) but positively correlated with age (r = 0.192, *P* < 0.01). The relationship between anti-N IgG S/C index and interval between symptom onset and blood sampling was plotted using the median and IQR. The regression analysis confirmed that IgG S/C index decreased over time. The patterns were similar in patients without (r^2^ = 0.321, *P* < 0.01) and with (r^2^ = 0.321, *P* < 0.01) pneumonia.

**Figure 5:**
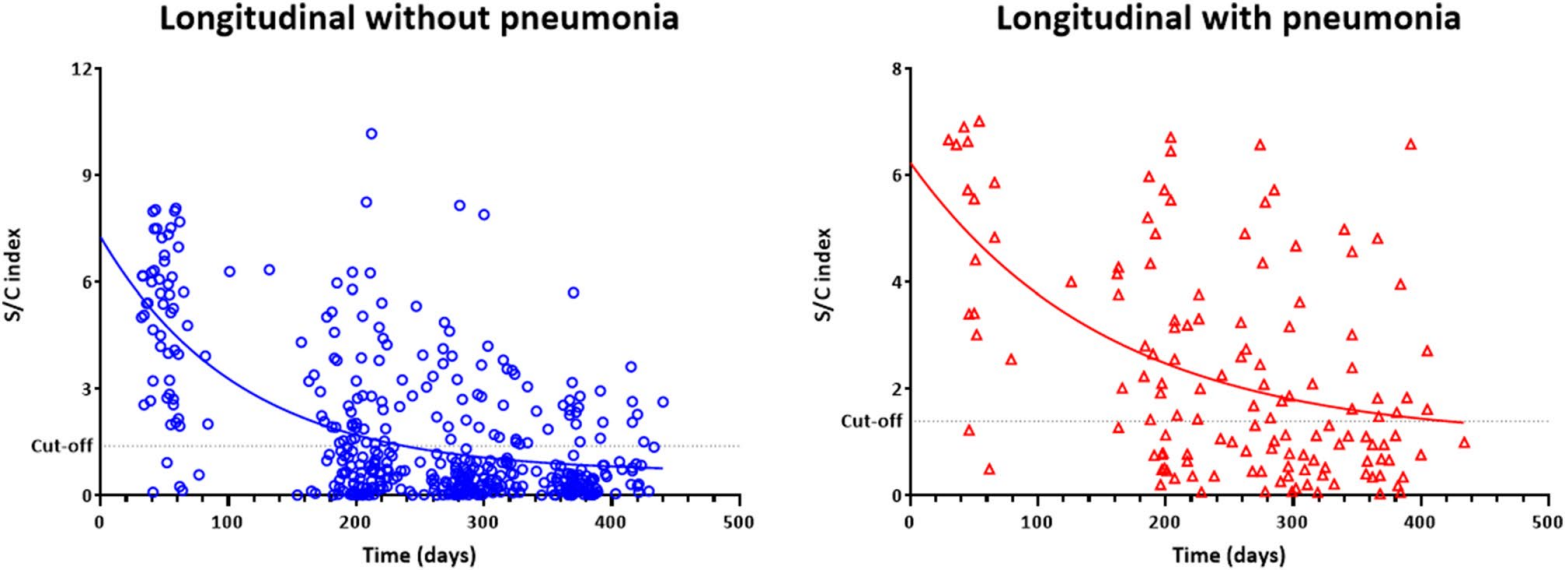
SARS-CoV-2–specific anti-nucleocapsid IgG S/C index in a longitudinal cohort of recovered COVID-19 patients who provided blood samples for at least three time points. (**a**) Patients without pneumonia. (**b**) Patients with pneumonia.

The anti-N IgG S/C index against SARS-CoV-2 in a longitudinal cohort of recovered COVID-19 patients who provided blood samples for at least three time points were plotted over time using median and IQR (Fig. 5). In total, there were 133 patients without pneumonia and 44 patients with pneumonia. The model predicted an anti-N IgG half-life of 75.4 days (95% confidence interval [95% CI]  51.7–112.0, R^2^ = 0.37) in the non-pneumonia group and 107.6 days (95% CI 39.4–974.5, R^2^ = 0.24) in the pneumonia group. No significant differences were observed in the decay kinetics between the two groups.

## Discussion

Knowledge of the durability of the immune response against SARS-CoV-2 is essential for predicting protection and herd immunity and interpreting serology and epidemiology data. The present study evaluated the seropositivity rate of anti-N IgG against SARS-CoV-2 in patients 3–12 months after the onset of COVID-19 symptoms. The results showed that > 60% of patients who had recovered from COVID-19 lost their detectable anti-N IgG at 6 months after symptom onset; at 9 and 12 months after natural infection, approximately one-quarter had detectable anti-N IgG.

Antibodies specific to the nucleocapsid protein of SARS-CoV-2 do not neutralize the virus but may still contribute to the immune control of infection through viral clearance by antibody-dependent cellular cytotoxicity^19^. The SARS nucleocapsid protein contains several T cell epitopes that stimulate the T cell response in a vaccine setting, inducing SARS-specific T cell proliferation and cytotoxic activity^20,21^. A longitudinal study on the persistence of IgG to SARS-CoV-2 nucleocapsid protein detected using quantitative assays showed that anti-N IgG titer declined a few months after symptom onset, which occurred more rapidly in younger adults and asymptomatic individuals^13^. However, a qualitative study that used optical density as a measure showed that 90% of patients still had detectable ant-N IgG 1 year after symptom onset^22^. Although our study used a qualitative measure (IgG S/C index), our results are in agreement with those obtained with the quantitative assay.

In the present study, COVID-19 patients in the pneumonia group tended to generate a higher antibody response than those in the non-pneumonia/asymptomatic group; seropositivity rates also remained significantly higher in the former at 6, 9, and 12 months after infection. Our findings are in agreement with a previous study which demonstrated that anti-N IgG was significantly higher and persisted longer in patients with severe conditions^23,24^.

The anti-N IgG S/C index declined over time with an approximate half-life of 75.4 days in the non-pneumonia/asymptomatic group and 107.6 days in the pneumonia group. We calculated half-life from the anti-N IgG S/C index between days after symptom onset in a longitudinal cohort of recovered COVID-19 patients who provided blood samples for at least three time points. The half-life of anti-N IgG was previously estimated as 68 and 71 days^25^. The half-life of antibodies is longer when assessed after as compared to before 3 months post symptom onset^25^. This is likely due to the rapid decline of antibody titer during the first few months post infection, after which the levels stabilize.

It was reported that persons who have had COVID-19 retained immune memory for at least 6 months^26^. Long-lived bone marrow plasma cells specific to SARS-CoV-2 have been detected in the bone marrow of recovered COVID-19 patients, serving as a persistent and essential source of antibodies^27^. A clinical study showed that reinfection was rare 1 year after primary infection, likely due to the protective effect of natural infection^28^. Nevertheless, there are limited data on whether protective immunity induced by natural infection with one strain of SARS-CoV-2 can confer cross-protection against variants. Because of the emergence of new variants and their circulation in the population, the Centers for Disease Control and Prevention recommends that the COVID-19 vaccine be given to recovered COVID-19 patients after 90 days post symptom onset. The World Health Organization has also stated that recovered COVID-19 patients can wait up to 6 months, during which time their natural immunity can protect them against reinfection.

This study had some limitations. Cross-sectional blood sampling may have prevented the detection of changes in immune response against SARS-CoV-2 compared to a longitudinal cohort. Additionally, analysis of other immune responses such as IgG against spike protein or spike receptor-binding domain, neutralizing antibodies, and memory B cells, can provide insight into the humoral response following natural infection, which can guide immunization strategies for patients who have recovered from COVID-19.

In summary, the results of this study demonstrate the generation of a persistent immunologic response to SARS-CoV-2 even after recovery from COVID-19; seropositivity was observed up to 12 months after symptom onset in one-quarter of previously infected patients. Additionally, the anti-N IgG S/C index was correlated with disease severity in patients. However, long-term monitoring is needed to determine whether this immunologic memory confers protection against reinfection with the same or a new variant of SARS-CoV-2.

## Methods

### Ethics statement

The study protocol was approved by the Research Ethics Committee of the Faculty of Medicine, Chulalongkorn University (Institutional Review Board [IRB] no. 572/63). The blood was drawn from participants in a research setting as part of a pre-planned follow up. Written informed consent was obtained from all participants prior to enrollment (IRB No. M001h/63_Exp, approved by the Institutional Ethics Committee of the Bangkok Metropolitan Administration, Thailand and IRB No. 11/2563 approved by National Blood Center, Thailand). This study was conducted in accordance with the principle of the Declaration of Helsinki and Good Clinical Practice (GCP) Guideline. All methods were carried out according to relevant guidelines and regulations.

### Participants and blood sampling

**Figure 6:**
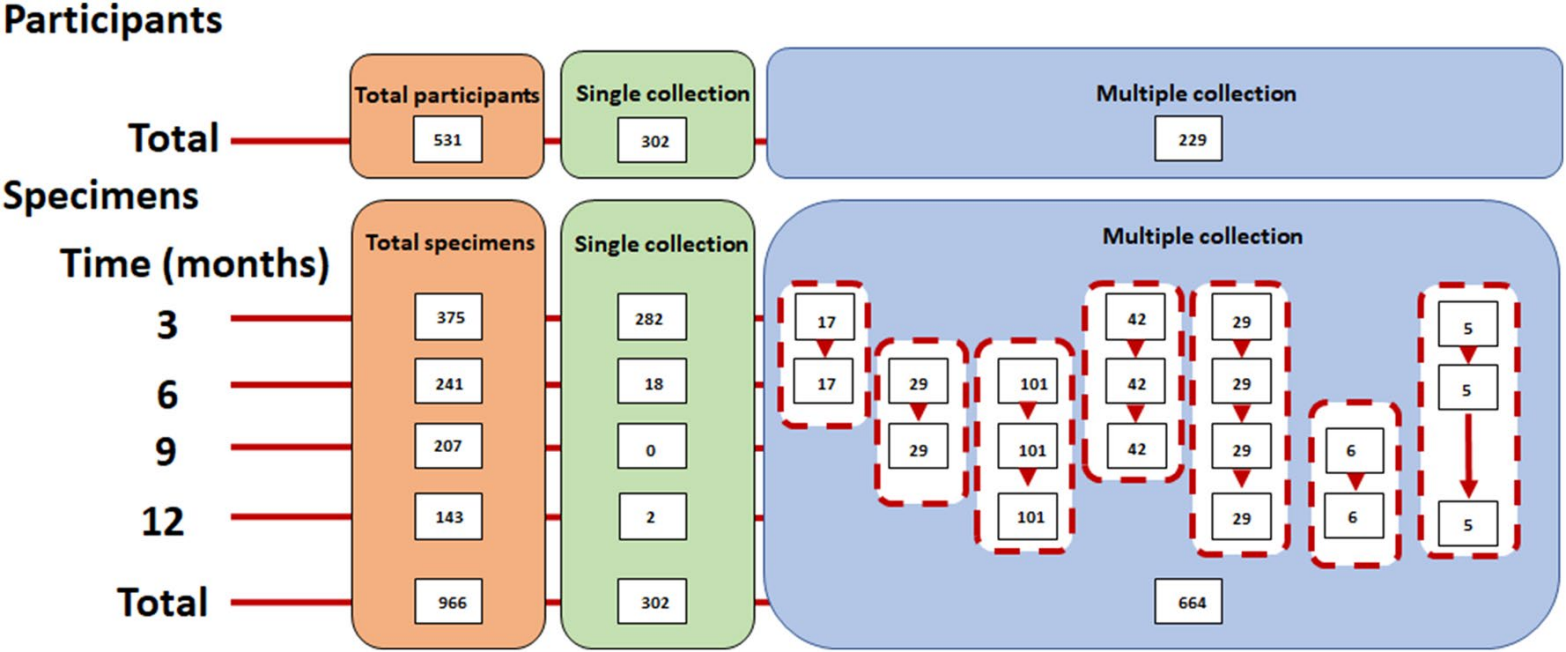
Flow diagram of participant recruitment and specimen collection in this study. A total of 531 participants were enrolled; 302 provided a blood sample at a single time point and 229 provided multiple samples.

This cross-sectional and longitudinal cohort study enrolled patients diagnosed with COVID-19 by RT-PCR (The cobas SARS-CoV-2 Qualitative assay for use on the cobas 6800/8800 Systems) between March 2020 and May 2020. Participants in this study were not in the immunocompromised state or had immunodeficiency disorders. There were 111 patients with pneumonia symptoms and 420 without these symptoms (non-pneumonia/asymptomatic group). Sample collection was performed 3 ± 1, 6 ± 1, 9 ± 1, and 12 ± 1 months after symptom onset or first detection of SARS-CoV-2 by RT-PCR in asymptomatic individuals. Patients’ age, sex, symptom severity (i.e., without or with pneumonia symptoms), and interval between symptom onset and date of blood sampling were recorded. A flow diagram of participant recruitment is shown in Fig. 6. A total of 302 participants provided blood samples at a single time point, while 229 provided multiple blood samples for 3–12 months. Presence or absence of pneumonia was determined retrospectively from the history taking at enrollment or patients’ medical records if available.

### Serologic testing

Blood samples were centrifuged for 10 min at 2000 rpm at room temperature. The supernatant (serum) was stored as aliquots in 2.0-ml tubes at − 20 °C until use. The specimens were tested for SARS-CoV-2 anti-N IgG by chemiluminescent microparticle immunoassay using the commercially available automated ARCHITECT system (Abbott Diagnostics, Sligo, Ireland), which calculates the mean chemiluminescent signal of a calibrator; the sample result is then divided by the stored calibrator result. The default unit for the SARS-CoV-2 anti-N IgG assay is the S/C index; S/C values ≥ 1.4 and < 1.4 were defined as positive and negative, respectively, according to the manufacturer’s instructions.

### Statistical analysis

The anti-N IgG S/C index of samples was plotted against interval between symptom onset and blood sampling using individual data and median with IQR. Graphs were generated using Prism v9.0 software (Graph Pad, San Diego, CA, USA). Statistical analyses were performed using SPSS Statistics for Windows v21 software (IBM, Armonk, NY, USA). The chi-squared test was used to compare the seropositivity rates between participants in different disease severity, interval between symptom onset and blood sampling, sex, and age groups. Pearson’s correlation coefficient was computed to assess the relationship between the anti-N IgG S/C index and interval between symptom onset and blood sampling. The regression/correlation between the anti-N IgG S/C index and interval between symptom onset and blood sampling was calculated by linear regression. A *p* value < 0.05 was considered significant in all tests.

## Supplementary Information

Supplementary Information.

## Data Availability

The authors confirm that the data supporting the findings of this study are available within the article.
