# Supplementary material for: Long-term specific IgG response to SARS-CoV-2 nucleocapsid protein in recovered COVID-19 patients
Source: Sci Rep. 2021 Dec 1;11:23216. doi: 10.1038/s41598-021-02659-4 (PMC8636620; doi:10.1038/s41598-021-02659-4)
Supplement: Supplementary file 1 — Supplementary Information. [file 41598_2021_2659_MOESM1_ESM.docx]

**Supplementary Table 1** Seropositivity rate of specimens in this study

| **Specimens** | **Characteristic** | **Symptoms** | | **Chi-squared (*P*-value)** |
| --- | --- | --- | --- | --- |
|  |  | **Without pneumonia,**  **N = 420** | **With pneumonia,**  **N = 111** |  |
| 3 Months post symptom onset at collection | Seropositivity (%) | 260/300 (86.7) | 68/75 (90.7) | 2.0 (0.4) |
|  | Median anti-N IgG S/C index (IQR) | 4.5 (2.7–6.1) | 5.7 (3.9–6.6) |  |
|  | GMT (95% CI) | 3.0 (2.6–3.4) | 4.2 (3.5–5.1) |  |
| 6 Months post symptom onset at collection | Seropositivity (%) | 61/188 (32.4) | 33/53 (62.3) | 18.3 (<0.01) |
|  | Median anti-N IgG S/C index (IQR) | 0.8 (0.2–1.9) | 1.9 (0.7–3.5) |  |
|  | GMT (95% CI) | 0.6 (0.5–0.8) | 1.3 (0.9–1.9) |  |
| 9 Months post symptom onset at collection | Seropositivity (%) | 31/158 (19.6) | 18/49 (36.7) | 6.3 (0.04) |
|  | Median anti-N IgG S/C index (IQR) | 0.5 (0.1–1.0) | 1 (0.5–2.5) |  |
|  | GMT (95% CI) | 0.4 (0.3–0.5) | 0.9 (0.6–1.3) |  |
| 12 Months post symptom onset at collection | Seropositivity (%) | 23/107 (21.5) | 15/36 (41.7) | 6.3 (0.04) |
|  | Median anti-N IgG S/C index (IQR) | 0.4 (0.1–0.9) | 1.1 (0.6–2.3) |  |
|  | GMT (95% CI) | 0.4 (0.3–0.5) | 1.0 (0.6–1.5) |  |
| Total specimens | Positivity (%) | 375/753 (49.8) | 134/213 (62.9) | 32.1 (<0.01) |

Abbreviations: CI, confidence interval; GMT, geometric mean titer; IgG, immunoglobulin G; IQR, interquartile range.
